# The Potential Role of MicroRNA-124 in Cerebral Ischemia Injury

**DOI:** 10.3390/ijms21010120

**Published:** 2019-12-23

**Authors:** Xiaolu Liu, Zhitao Feng, Lipeng Du, Yaguang Huang, Jinwen Ge, Yihui Deng, Zhigang Mei

**Affiliations:** 1 Third-Grade Pharmacological Laboratory on Chinese Medicine Approved by State Administration of Traditional Chinese Medicine, Medical College of China Three Gorges University, Yichang 443002, China; sharonliu2017@163.com (X.L.); fengzhitao2008@126.com (Z.F.); dulipeng1994@163.com (L.D.); huyagu@163.com (Y.H.); 2 The Key Laboratory of Hunan Province for Integrated Traditional Chinese and Western Medicine on Prevention and Treatment of Cardio-Cerebral Diseases, Hunan University of Chinese Medicine, Changsha 410208, Chinadengyihui06@126.com (Y.D.)

**Keywords:** microRNA-124, cerebral ischemia, ischemic encephalopathy, ischemic stroke, neurodegenerative disease

## Abstract

Cerebral ischemia injury, the leading cause of morbidity and mortality worldwide, initiates sequential molecular and cellular pathologies that underlie ischemic encephalopathy (IE), such as ischemic stroke, Alzheimer disease (AD), Parkinson’s disease (PD), epilepsy, etc. Targeted therapeutic treatments are urgently needed to tackle the pathological processes implicated in these neurological diseases. Recently, accumulating studies demonstrate that microRNA-124 (miR-124), the most abundant miRNA in brain tissue, is aberrant in peripheral blood and brain vascular endothelial cells following cerebral ischemia. Importantly, miR-124 regulates a variety of pathophysiological processes that are involved in the pathogenesis of age-related IE. However, the role of miR-124 has not been systematically illustrated. Paradoxically, miR-124 exerts beneficial effects in the age-related IE via regulating autophagy, neuroinflammation, oxidative stress, neuronal excitability, neurodifferentiation, Aβ deposition, and hyperphosphorylation of tau protein, while it may play a dual role via regulating apoptosis and exerts detrimental effects on synaptic plasticity and axonal growth. In the present review, we thus focus on the paradoxical roles of miR-124 in age-related IE, as well as the underlying mechanisms. A great understanding of the effects of miR-124 on the hypoxic–ischemic brain will open new avenues for therapeutic approaches to protect against cerebral ischemia injury.

## 1. Introduction

Cerebral ischemia, one of the leading causes of morbidity and mortality worldwide, is characterized by insufficient blood supply to the brain with subsequent insufficiency in the delivery of oxygen and nutrients, which may trigger anatomic alteration, such as increased blood–brain barrier (BBB) permeability [1] and a vast array of pathological processes including apoptosis, inflammation, excitotoxicity, oxidative stress, and mitochondrial dysfunction that lead to neuronal cell death [2,3], hindering important sensory [4], motor [5], and cognitive functions [6]. Cerebral ischemia has been found to exist as a major contributor to the dysfunction in the aging brain and the pathological conditions of neurodegenerative diseases [7], such as ischemic stroke [8], Alzheimer’s disease (AD) [9], Parkinson’s disease (PD) [10], and epilepsy [11]. Several classical pathological processes associated with cerebral ischemia, including apoptosis [12], inflammation [13], and oxidation stress [14], have been elaborated in the past decades, newly characterized mechanisms are emerging with the development of systems biology in areas like genomic, transcriptomics, and proteomics have brought cerebral ischemia to a new level. Bioinformatic analysis reveals that microRNAs (miRNAs) are altered after focal cerebral ischemia and target the translation of proteins that modulate inflammation [13], autophagy [15], apoptosis [16], and oxidative stress [17] in age-related ischemic encephalopathy (IE).

MiRNAs, members of the small noncoding RNA superfamily, are endogenous single stranded RNA molecules of approximately 18–25 nucleotides, which function as negative regulators of the expression of over 60% of protein-coding genes by degradation or translation inhibition of target mRNA [18]. MiRNAs are able to simultaneously regulate the targets that participate in pathophysiological processes of cerebral ischemia, and are considered as potential diagnostic and prognostic biomarkers, as well as promising therapeutic agents in cerebral ischemic stroke [19]. MicroRNA-124 (miR-124), a preferentially expressed miRNA in the cerebral cortex and cerebellum, is initiated at a low level in neural progenitors and reaches a high level in differentiating and mature neurons [20,21], suggesting an important role for this molecule in neuronal development. Furthermore, miR-124 is also abundantly expressed in microglia [22]. Research has revealed that upregulation of miR-124 could regulate apoptosis and impaired autophagy process in PD, thus reducing the loss of dopaminergic neurons [23]. Furthermore, miR-124 has also been identified as a key regulator of microglia quiescence in the central nervous system (CNS), as well as a modulator of monocyte and macrophage activation [22]. Although it is not expressed into astrocytes [24], miR-124 can be transferred from neurons to astrocytes via neuronal exosomes, which then selectively upregulate the expression of glutamate transporter 1, one of the most important astroglial functional synaptic proteins [25]. Its abundance in the adult brain of a variety of mammalian species ranges from 25% to 48% of all miRNAs expressed, and miR-124 plays a critical role in controlling neuronal differentiation [21], neuroimmunity [26], synaptic plasticity, and axonal growth [27], which exerts fundamental effect in normal brain processes, as well as neuropathological conditions including AD, PD, and epilepsy [28]. Emerging studies demonstrated the neuroprotective effect of miR-124 in CNS diseases, such as brain tumors [29] and neuroimmune disorders [30]. In addition, it has been reported that miR-124 is capable to protect neurons in cerebral ischemia via regulating key genes [31] and pathological processes, which consist of autophagy [23], neuroinflammation [13], oxidative stress [17], neuronal excitability [32], neurodifferentiation [33], Aβ deposition [34], and hyperphosphorylation of tau protein [35]. However, miR-124 also exerts dual effects via regulating apoptosis in cerebral ischemia [36]. In this review, we will give insight into the role of miR-124 as a potential and novel diagnostic and prognostic biomarker, with particular focus on the therapeutic effects on IE.

## 2. MiR-124 in Ischemic Encephalopathy

**Figure 1:**
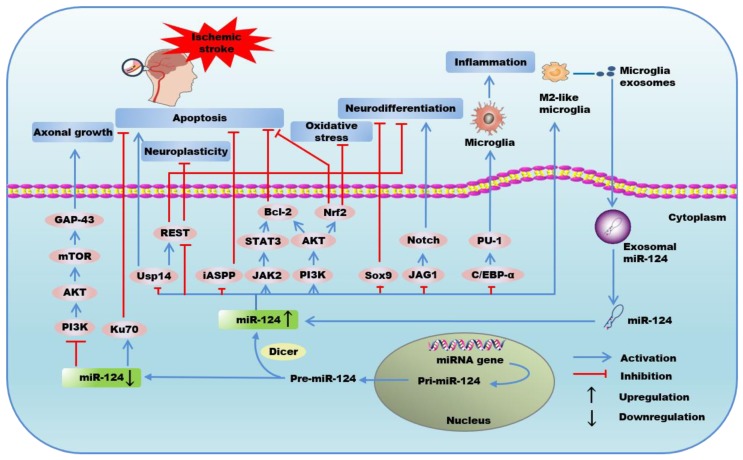
The possible mechanisms of the regulation of microRNA-124 in ischemic stroke.

Cerebral ischemia has been found to give rise to neurological dysfunction and degeneration disease. It has been revealed that dysregulation of miR-124 expression is not only linked to post ischemic neuronal survival and pathogenesis of cerebral ischemia via mechanisms involving protein degradation (Figure 1), but also demonstrated to possess promising potential for the diagnosis and treatment of age-related IE, and predict the prognosis of ischemic stroke. In the following paragraphs, we will summarize the pathophysiological mechanisms concerning miR-124 and IE, as well as the potential of miR-124 to be a diagnosis and prognosis biomarker and pharmacological target of age-related IE (Table 1).

### 2.1. MiR-124 and Ischemic Stroke

#### 2.1.1. Ischemic Stroke

Ischemic stroke is the most common cerebral ischemia disease and the second leading cause of death worldwide, accounting for 11.8% of total deaths, significantly threatening human health and life with a high disability and mortality rate [56]. Ischemic stroke is characterized by thromboembolic occlusion of a major artery and interruption of blood supply to the brain with subsequent insufficiency in the delivery of oxygen and nutrients [8], which cause neuronal cell death, abnormal synaptic activity, and neurological deficits, such as learning, memory, and locomotor deficiencies [57]. Ischemic stroke can lead to necrosis or neuronal survival by maintaining cellular homeostasis [58], and survival neurons after ischemic stroke undergo numerous modifications, including synaptic remodeling and axon growth, which are essential to spontaneous recovery [59]. A series of complex pathological mechanisms consisting of oxidative stress, excitotoxicity, inflammation, and apoptosis are responsible for post-ischemic brain injury [2,3]. Of note, plasma miR-124 has been demonstrated to decrease rapidly within 24 h after ischemia and gradually increase within 48–72 h, as well as the seventh day after ischemia in patients with ischemic stroke [60], and miR-124 also participates extensively in the pathophysiology following ischemic stroke, functioning as a protector against cerebral ischemia reperfusion injury (CIRI) [61], suggesting miR-124 as a promising candidate biomarker for the time-sensitive diagnosis and therapeutic target of ischemic stroke.

#### 2.1.2. Mechanisms of miR-124 in Ischemic Stroke

In light of the existing clinical and animal studies, aberrant plasma concentrations and serum exosomes of miR-124 were reported in ischemic stroke, which were correlated with infarct volume [62,63]. The changes in plasma concentrations of miR-124-3p in the early stage of ischemic stroke (<6 h) can predict three-month morbidity and mortality [64]. Moreover, the *visinin-like protein-1* (*VSNL-1*) gene, a neuronal calcium sensor protein identified as a specific plasma biomarker of stroke patients, was decreased and correlated with the increase of miR-124 expression following cerebral ischemia [65]. Additionally, upregulating the peripheral serum expression of miR-124 and downregulating laminin and integrin β1 levels by acupuncturing at Baihui (GV20) and Zusanli (ST36) intervened in CIRI could result in a reduction of the brain infarction and an improvement in neurological scores [61]. Moreover, the overexpression of miR-124 has been also observed to downregulate the expression of cyclin-dependent kinase family 4, which was involved in the pathogenesis of post-stroke motor function recovery [66]. These studies suggest a broad opportunity for developing the use of circulating miR-124 as a diagnostic and prognostic molecular for ischemic stroke and post-ischemic stroke outcomes, as well as a therapeutic target against ischemic stroke injury.

The anoxic environment of ischemic stroke stimulated the upregulation of miR-124, which was found to be a determinant of neuronal differentiation, neurovascular remodeling, neurite outgrowth, and synaptogenesis after ischemic stroke [39]. A previous study has reported that miR-124 is implicated in the positive modulation of adult subventricular zone neuronal differentiation by post-transcriptional downregulating the expression of the transcription factor Sox9, which is required for the generation and maintenance of adult subventricular zone neural stem cells [24]. Saraiva et al. found that intracerebroventricular delivery of miR-124-loaded nanoparticles (NPs) boosted neuronal differentiation of subventricular zone neural stem cells after oxygen and glucose deprivation (OGD) [67], and these newly generated neurons could replenish lost neurons and thereby contribute to synaptogenesis and behavioral recovery after a stroke [68]. The inactivation of a Notch signaling pathway has been revealed to enhance neuronal differentiation in ischemic neural progenitor cells [69]. Liu et al. found that the introduction of biological simulants of miR-124a into ischemic neural progenitor cells significantly inhibited ischemia-induced neuronal progenitor cells proliferation, while promoted progenitor neurons differentiation via targeting and repressing jagged-1 (JAG1), a ligand of the transmembrane protein Notch, leading to inactivation of Notch signals [33]. The upregulated expression of miR-124 facilitates RE1-silencing transcription factor (REST) degradation, which promoted post-ischemic stroke neuroplasticity and angiogenesis, expanding the evidence that miR-124 might present a promising target [44]. Additionally, Doeppner et al. reported that application of miR-124 could directly target and suppress deubiquitinating enzyme Usp14, thereby mediating the decrease of REST, leading to reduced ischemic injury by enhancing neuronal differentiation and neurovascular remodeling [37]. However, Zhu et al. revealed that the inhibition of miR-124 could activate PI3K/AKT/mTOR signaling pathway and then upregulate growth associated protein-43 (GAP)-43 expression after OGD/R, leading to the promotion of axonal growth, which promoted contacting with surrounding cells and transmission of information and nutrient delivery [39]. In summary, whether miR-124 exhibits the therapeutic or detrimental profile in ischemic stroke remains obscure and is worthy of further researching.

Post-ischemic inflammation, a pivotal pathophysiological mechanism in ischemic stroke, can trigger damaged tissue and necrotic cells [13]. An initial inflammatory event in ischemic stroke is the activation of microglia cells, the first line of the innate immune response in CNS and is activated a few minutes after ischemic stroke onset [70]. At the early stage of ischemic stroke, microglia cells possess anti-inflammatory effects (M2 phenotype), while at the later stage, microglia cells with the pro-inflammatory M1 phenotype secrete inflammatory cytokines, such as necrosis factor-α (TNF-α), toll-like receptor 4, and matrix metalloproteinase 9 [71,72], which are involved in the leakage of the blood–brain barrier. Therefore, suppressing the over-reaction of microglia and microglia-mediated neuroinflammation is deemed to be a therapeutic strategy for cerebral ischemia-induced damage. Hamzei et al. found that the intracerebral injection of miR-124 suppressed development of inflammation by skewing the microglia into the M2 anti-inflammation phenotype after ischemic stroke [13], which could release anti-inflammatory factors such as interleukin (IL)-10, IL-4, and IL-13, transforming growth factor β, and activating T regulatory cells [73], subsequently improving tissue repair and long-term neurological outcomes after ischemic stroke. Ning et al. reported that miR-124 was at least partially contributing to the apolipoprotein-A1 mimetic peptide, D-4F, induced M2 macrophage polarization and anti-inflammatory effects in type one diabetes mellitus (T1DM) ischemic stroke rats, reducing the expression of inflammatory factors in the ischemic brain, and improving neurological functional outcomes after ischemic stroke [72]. Additionally, activated microglia can release cytokines and cytotoxic factors that accelerate the occurrence or deterioration of neurodegenerative diseases [74], and promote the proliferation of astrocytes, glial fibrillary acidic protein, and some other cytokines that contribute to the enhancement of the glia scar, which has major effects on neuronal excitability and secondary pathologies, such as epilepsy [75,76]. Moreover, overexpression of miR-124 has been found to promote quiescence of microglia and deactivation of macrophages by directly targeting and inhibiting the expression of transcription factor CCAAT/enhancer-binding protein alpha (C/EBP-α), which leads to downregulation of downstream target PU.1 [22]. These results identify miR-124 as a novel anti-inflammatory regulator that could contribute to the treatment of age-related IE.

Apoptosis is a principle mechanism that participates in neural loss in the ischemic region, and miR-124 exhibits dual effects on neurons by regulating apoptosis. Wang et al. observed that miR-124 exerted an anti-apoptosis effect in ischemic stroke via activating phosphoinositide 3-kinase (PI3K)/protein kinase B (PKB/AKT) signaling pathway, a well-established signaling pathway positively affected expression of B-cell lymphoma-2 (Bcl-2), further alleviated cell apoptosis in ischemic stroke [40]. Additionally, miR-124 could also protect PC12 cells against OGD/R-induced injury by inhibiting oxidative stress and apoptosis, which was associated with increasing heat-shock protein expression through the activation of PI3K/AKT/Nrf2 pathway [41]. MiR-124 could also downregulate the expression of inhibitory member of the apoptosis-stimulating proteins of p53 family (iASPP) in the early stage of ischemic stroke, suppressing this endogenous pro-survival pathway and promoting neuronal apoptosis after ischemic stroke, while the inhibition of miR-124 upregulated the level of iASPP and effectively decreased infarct volume [36]. Sun et al. reported that the level of miR-124 was significantly increased in ischemic penumbra and upregulation of miR-124 protected neurons against apoptotic cell death in ischemic stroke by increasing anti-apoptosis proteins, Bcl-2 and Bcl-xl, respectively [42]. Studies have confirmed that cerebral ischemia/reperfusion (I/R) promotes the phosphorylation of Janus tyrosine kinase 2 (JAK2), and its downstream signal transducer and activator of transcription 3 (STAT3), which exerts an anti-apoptosis effect and promotes cell viability [77]. Moreover, miR-124 exhibited protective effects against CIRI through inhibiting apoptosis via activating JAK2/STAT3 signaling pathway and its downstream anti-apoptosis protein Bcl-2 [16], while Zhu et al. observed that knockdown of miR-124 in vivo led to the improvement of brain injury and neuronal outcomes against I/R-induced neuronal death and apoptosis via directly targeting and upregulating Ku 70 mRNA and protein expression [43], which was able to inhibit Bax-mediated apoptosis by blocking the mitochondrial translocation of Bax [78]. All of these studies suggest that the intervention of miR-124 might be a significant therapeutic target to address ischemic stroke or I/R-induced apoptosis.

#### 2.1.3. Exosomal miR-124 in Ischemic Stroke

Drug delivery to the brain for the treatment of neurological diseases is hard to implement because the BBB blocks many drugs, while exosomes, the lipid membrane vesicles of 30–100 nm in diameter, are secreted by most cell types and capable of crossing BBB [79], which provides an incentive to develop these membrane vesicles into potential forms of drug carriers. Exosome-mediated intracellular communication can contribute to angiogenesis and neurogenesis by the delivery of biologically active molecules, such as nucleic acids, mRNAs, and miRNAs from source cells to recipient cells [80,81], and neuro-secreted exosomes have been found to contribute to local synaptic plasticity after ischemic stroke [81]. Recently, the exosomal miR-124 has emerged as a novel diagnostic biomarker and therapeutic target for CNS diseases, such as Huntington’s disease [82] and ischemic stroke [83]. The serum exosomal miR-124 is significantly raised in acute ischemic stroke patients and positively correlates with the infarct volumes and brain damage degree [63]. High-concentration glutamic acid (Glu) induced by oxygen-glucose deprivation/reperfusion can result in the phosphatidylinositol signaling pathway coupled with Gq protein and ultimately induces cytotoxic edema, necrosis, and apoptosis in CNS. However, miR-124 can be transferred from neurons into astrocytes through neuronal exosomes, and then significantly upregulate astroglial glutamate transporter 1 (GLT1) in an indirect manner [25], suggesting a role of exosomal miR-124 as a regulator of astrocyte functions after ischemic stroke. Yang et al. reported that the delivery of rabies virus glycoprotein-exosomes loaded with miR-124 to infarct site could greatly promote progenitor cells toward neuronal lineage and reduce ischemic cortical injury after ischemic stroke [83]. Exosomal miR-124 derived from M2 microglia has been found to be taken up by neurons and exert neuronal protective effects in ischemic stroke through attenuating neuronal apoptosis via downregulating Usp14 [38]. These studies reveal that the serum exosomal miR-124 is a promising biomarker for diagnosing and evaluating the degree of damage caused by ischemic stroke, as well as a beneficial target for the treatment of ischemic stroke, and exosomes possess the potential to function as novel vehicles for the delivery of drugs into brain.

In summary, miR-124 exerts dual effects in ischemic stroke. MiR-124 can promote neuronal differentiation, neuroplasticity, and angiogenesis through targeting and inhibiting Sox9 [24], JAG1 [33], and Usp14 [37], while the negative constraint that miR-124 imposes on axonal growth is mediated by regulating PI3K/AKT/mTOR/GAP-43 signaling pathway [39]. Furthermore, miR-124 can alleviate inflammatory response after ischemic stroke via contributing to resting phenotype of microglia [22] and M2 macrophage polarization [73]. In addition, miR-124 suppresses apoptosis in ischemic stroke via directly targeting and upregulating Bcl2, Bcl-xl [42], Usp14 [38], JAK/STAT3 [77], and PI3K/AKT/Nrf2 signaling pathways [41], while other researchers reported that miR-124 promotes neuronal apoptosis through inhibiting iASPP [36] and Ku70 [43] in ischemic stroke (Figure 1).

### 2.2. MiR-124 and AD

#### 2.2.1. Alzheimer’s Disease 

AD, a predominantly amnestic dementia, has been suggested to be a form of neuroplasticity failure [84], featuring by progressive cognitive decline and neurodegeneration of brain regions that are crucial for learning and memory [85]. The extracellular excessive deposition of β-amyloid (Aβ) plaques, intracellular formation of neurofibrillary tangles (NFTs) composed of tau amyloid fibrils are considered as the main pathological features of AD [86]. Cerebral hypoperfusion has been found to contribute to the pathological features of AD, including upregulation of beta-site amyloid precursor protein cleaving enzyme1 (BACE1) [87], aggregation of Aβ [88], and hyperphosphorylation of tau [89], which can lead to synaptic dysregulation, deficits of progressive spatial memory, and neuronal loss in AD [90]. Moreover, cerebral ischemia can trigger endoplasmic reticulum stress and impair autophagy, which then stimulate the expression of presenilin 1. Subsequently, presenilin 1 stabilizes the expression of hypoxia-inducible factor-1α, which can stimulate the transcription of the *BACE1* gene through the hypoxia-response element in the *BACE1* promoter [91], giving rise to the formation of Aβ [92]. The level of miR-124 was downregulated in AD brain, which was involved in negatively regulating BACE1 expression and accumulation of Aβ [93], which indicates the miR-124 expression has the potential to generate novel diagnostic and therapeutic strategies to AD.

#### 2.2.2. Mechanisms of miR-124 in Alzheimer’s Disease 

Aβ plaques-mediated neurotoxicity has been shown to cause deficits in hippocampal synaptic plasticity and synapse loss [94], as well as blocking the persistence of long-term potentiation in the hippocampus, which would give rise to cognitive and memory impairment in patients with AD [95]. The exogenous Aβ, which is used to induce a cellular AD model, can lead to 86% cell death in primary cultured hippocampal neurons [93]. Impairment of Aβ clearance appears to be responsible for the abnormal Aβ aggregation and plaques formation in AD [96]. Feng et al. reported that the decreased expression of miR-124 in BV2 microglia cell upregulated regulatory factor X1 (RFX1) level, while decreased the expression of *apolipoprotein E (ApoE)* [34], a major apolipoprotein carrier in the brain that has been found to be a target gene of RFX1 and enhance Aβ degradation in microglial cells [97]. These studies suggest that targeting the association of miR-124 with *ApoE* might be a potential therapeutic strategy to improve Aβ clearance.

Exposure of rat hippocampal neurons to Aβ decreases the expression of *cAMP-response element-binding protein (CREB)* and other memory-related genes including *brain-derived neurotrophic factor (BDNF)* [98,99]. However, synaptic efficacy mediates memory storage and requires specific gene expression programs regulated by the transcription factor CREB [100]. The inhibition of miR-124 has been found to result in a robust upregulation of CREB, which is known as a central transcriptional mediator of neuronal response to BDNF [101] and a crucial activator of transcription required for long-term plasticity of synapses [102]. Of note, rescuing impaired synaptic plasticity has been considered to contribute to restoring the memory deficits in AD [103]. MiR-124 is exclusively present presynaptically in a sensory-motor synapse and functions as a negative constraint on 5-hydroxytryptamine dependent long-term facilitation and synaptic plasticity via inhibiting the translation of CREB by directly binding CREB 3’UTR [27]. Furthermore, 5-hydroxytryptamine has been well established to regulate synaptic protein and memory enhancement via miR-124/CREB signaling pathway [104]. The studies above have suggested a crucial role for miR-124 in long-term plasticity of synapses. Zhao et al. reported that resveratrol could significantly improve the impaired memory formation and synaptic plasticity in AD via downregulating the miR-124 level, which is accompanied with an increase of CREB and subsequently promoted BDNF synthesis [45], a key molecule in the neurobiological mechanisms relates to dendritic spine size and cognitive decline and also possesses neuroprotective properties against Aβ-induced neurotoxicity in cultured neurons and AD mouse models [105]. In addition, hyperoxygenation treatment has been found to revitalize AD pathology via increasing neurotrophic factors including BDNF [106]. However, miR-124 has been found to result in a significant downregulation of cocaine-induced plasticity by targeting and suppressing BDNF [107]. MiR-124/tyrosine-protein phosphatase non-receptor type 1 (PTPN1) pathway has also been reported to mediate synaptic plasticity and memory deficits in AD. Wang et al. found that the miR-124 level dramatically increased in AD hippocampus, which was accompanied with the decreased expression of its target, PTPN1. The overexpression of miR-124 or knockdown of PTPN1 could induce AD-like phenotypes, including deficits in synaptic transmission and plasticity, while disrupting the miR-124/PTPN1 interaction could alleviate the synaptic failure and memory deficits in AD [46]. These findings give evidence of the value of miR-124 as novel therapeutic target against AD, exerting impact on enhancing synaptic plasticity and improving cognitive deficits.

BACE1, the rate-limiting protease in the production of Aβ, is required to give rise to Aβ from the membrane-spanning Aβ precursor protein, which plays a crucial role that triggers the pathological cascade and results in neurodegenerative events [92]. Therefore, BACE1 is one of the prime therapeutic targets for blocking the early pathologic events in AD. More importantly, miR-124/BACE1 axis has been considered as a novel target in AD treatment [108]. An et al. observed that miR-124 was a negative regulator of BACE1 by directly targeting the 3’UTR of BACE1 mRNA, and the downregulation of miR-124 attenuated Aβ-induced apoptosis and cell viability inhibition in SH-SY5Y cells [47]. Furthermore, Fang et al. found the expression of BACE1 could be upregulated on the consequence of the downregulation of miR-124, and further exacerbated amyloid pathology, resulting in the promotion of endogenous Aβ production and Aβ neurotoxicity-induced cell death [93]. These reports suggest that miR-124 may function as an important candidate inhibitor of the development of AD via inhibiting secretase activity of BACE1.

Aβ can upregulate the generation of NFTs through Glycogen synthase kinase 3 (GSK)-3 activation, resulting in the phosphorylation of tau [109], which is more impactful on cognitive and functional decline than Aβ in AD mice [110]. Evidence suggested that hyperforin could decrease the hyperphosphorylation of tau and NFTs proteins through activating AKT and inhibiting GSK-3β activity via increasing the phosphorylation of GSK-3β, a major substrate of AKT, exerting protective effects in AD [111]. MiR-124-3p, a subtype of miR-124, was markedly decreased in AD. However, transfection of miR-124-3p mimics exerted a protective role in AD by attenuating the abnormal hyperphosphorylation of tau protein and tau-induced cell apoptosis through directly targeting and repressing Caveolin-1 expression, which promoted the activation of PI3K/AKT and phosphorylated GSK-3β, subsequently inhibited the activity of GSK-3β [35]. Moreover, miR-124 has been found to be upregulated by alpha 7 nicotinic acetylcholine receptor (α7nAChR) and exerted GSK3β-induced neurotoxic inhibitory effects, leading to improved impulsive and anxiety control in AD [112]. Results from these findings highlight the impact of miR-124 on phosphorylation of tau protein through regulating PI3K/AKT/GSK-3β signaling pathway and the inhibitory effects on GSK3β, which may provide a novel therapeutic avenue in AD.

**Figure 2:**
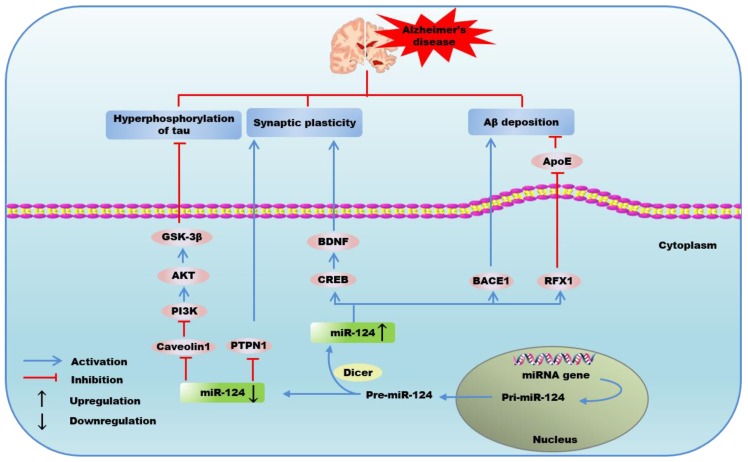
The possible mechanisms of the regulation of microRNA-124 in Alzheimer’s disease.

The critical role of miR-124 in Aβ production, synaptic/memory dysfunction, and phosphorylation of tau in AD suggests a potential novel therapeutic strategy for AD patients. Downregulation of miR-124 is able to upregulate RFX1 [34] and BACE1 [93], respectively, in AD, which participate in Aβ degradation and Aβ production. In addition, miR-124 can modulate CREB–BDNF axis [45] and PTPN1 expression [46], which are responsible for dendritic spine size and synaptic plasticity, suggesting the modulation of miR-124 may be beneficial for preserving cognitive function in AD. Furthermore, miR-124-3p plays a protective role by inhibiting abnormal hyperphosphorylation of tau protein, a pathological event occurs in the early stage of AD, via regulating Caveolon1-PI3K/AKT/GSK-3β [35] (Figure 2).

### 2.3. MiR-124 and PD

#### 2.3.1. Parkinson’s Disease 

Parkinson’s disease, an age-associated neurodegeneration disorder, ranks the second most common neurodegenerative disorder after AD and is predicted to increase in prevalence as the population ages [113]. The pathogenesis of PD has been proposed to associate with mitochondrial disturbance, oxidative stress, apoptosis, and inflammatory response of microglial cells [114]. Progressive degenerative loss of dopamine neurons in the substantia nigra pars compacta (SNpc) within the midbrain and formation of intracytoplasmic proteinaceous inclusions known as Lewy bodies (misfolded α-synuclein) constitute basic pathological hallmarks of PD [115]. The loss of nigrostriatal dopaminergic neurons results in dopaminergic deafferentation of the basal ganglia, giving rise to progressive deterioration of the motor function, coupled with the impairment of autonomic and cognitive functions [116]. Chronic cerebral hypoperfusion was observed as an independent exacerbating factor to the pathopoiesis of PD, and the extent of cognitive impairment corresponds to the degree of cerebral microvascular deficits [117]. A significant loss of dopaminergic neurons is observed after cerebral ischemia [118]. Additionally, the cellular environment after cerebral ischemia, such as oxidative stress and inflammation, potentially provide an optimal condition for α-synuclein aggregation [119], which plays a key role in neuronal toxicity by impairing a variety of cellular processes, leading to an overall decrease in motor performance [120]. At present, the diagnosis of PD is made based on identification of motor symptoms when a considerable number of dopaminergic neurons are already lost. Evidence demonstrates that the plasma level of miR-124 is significantly lower in patients with PD [121], revealing a promising biomarker for the diagnosis of PD.

#### 2.3.2. Mechanisms of miR-124 in Parkinson’s Disease 

Increasing evidence has pointed out that apoptosis and autophagy contribute significantly to neuronal loss in PD [122]. Bax, a pro-apoptotic factor of the Bcl-2 family, has been found to increase in SNpc of 1-methyl-4-pheny-1,2,3,6-tetrahydropyridine (MPTP)-treated mice, and the ablation of Bax can alleviate SNpc neuronal apoptosis [123]. Wang et al. found that exogenous delivery of miR-124 could maintain the number of dopaminergic neurons and midbrain dopamine level in PD model via directly targeting and inhibiting Bim, a bcl-2 homology-3-only protein that exerts apoptosis-inactive and autophagy-inhibitory effects by reducing Bax translocation to mitochondria and lysosomal membrane under stimulation of methyl phenyl pyridinium (MPP) [23]. Research showed that PD stresses led to the activation of adenosine 5’-monophosphate-activated protein kinase (AMPK) and inactivation of AKT, causing neuronal cell death via inhibiting mTOR [124]. More importantly, the downregulated miR-124 in MPTP-treated neurons could induce neurons apoptosis and autophagy-associated protein expression via activating p-AMPK, and then led to the inhibition of p-mTOR signaling, while the upregulation of miR-124 acted as a protector of dopamine neurons by suppressing cell apoptosis and autophagy through AMPK/mTOR signaling pathway in PD [48]. Geng et al. observed that the overexpression of miR-124-3p exerted a neuroprotective action in PD by alleviating neuronal injury via dramatically suppressing the activity of STAT3 through directly targeting the 3’UTR of STAT3 mRNA, attenuating MPP iodide-induced apoptosis, neuroinflammation, and oxidative stress [49]. Moreover, Dong et al. identified miR-124-3p as a neuronal protector by targeting and suppressing annexin 5 (ANAX5)/extracellular regulated protein kinases (ERK) signaling pathway, subsequently ameliorated 6-OHDA-induced neurotoxicity via alleviating ROS accumulation-mediated Caspase-3 activation, and apoptosis in PD [50]. Metastasis-associated lung adenocarcinoma transcript 1 (MALAT1), a regulator of gene expression that is involved in the dendritic and synapse development, can directly bind to miR-124 and negatively regulate its expression. Liu et al. demonstrated that MALAT1 knockdown inhibited apoptosis by conspicuously reversing MPTP/MPP induced miR-124 suppression, which further restrained the increase of caspase-3 in an in vivo and in vitro model of PD [51]. Thus, the mechanisms underlying the neuroprotection of miR-124 on PD is partially owed to the alleviation of apoptosis and autophagy.

Inflammatory reaction mediated by microglia cells plays a crucial role to accelerate neuronal injury in the pathological progression of PD [114]. Yao et al. identified a unique protective role of the exogenous delivery of miR-124, which could effectively attenuate the activation of microglial in the substantia nigra pars compacta of MPTP-induced PD model via targeting and suppressing mitogen-activated protein kinase kinase kinase 3 (MEKK3)/NF-kB signaling pathway [52]. Moreover, miR-124 could also target and downregulate the expression of sequestosome 1 (p62) and phosphor-p38 mitogen-activated protein kinase (p-p38), suppressing the activation of microglia and downregulating inflammatory response in PD [53].

The neurotoxicity induced by α-synuclein is partially through the activation of CDK5, a key kinase of CNS required for regulating cellular events in neuronal diseases [125], which can effectively participate in controlling inflammasome activation and oxidative stress in PD progression as well [17,126]. Notably, the downregulation of miR-124 in SNpc dopamine neurons of a MPTP-intoxicated PD mouse model has been found to modulate the expression of calpain1/CDK5 pathway proteins [127]. Kanagaraj et al. found that overexpression of miR-124 diminished the production of ROS and hydrogen peroxide (H_2_O_2_) via inhibiting calpain1/p25/CDK5 pathway, leading to the improvement of cell viability and survival in MPP-treated MN9D dopaminergic neurons [17]. Studies suggested that CDK5 was also implicated in neurodegenerative diseases. Moreover, synaptic failure represents the major pathogenic determinant in PD, and reflects the degree of biochemical impairment at the early stage [128]. Johana et al. demonstrated that CDK5 silencing could give rise to an upregulation of BDNF and Tropomyosin Receptor kinase B, subsequently activating the downstream cascade proteins ERK and CREB in cerebral ischemia-induced neurodegeneration and motor dysfunction [129], suggesting a gene therapy to cerebral ischemia-related neurodegenerative diseases based on CDK5 silencing. These studies provide evidence to support a potential mechanistic role for miR-124 to regulate the inflammatory pathogenesis and synaptic plasticity in PD, which may be associated with the expression of CDK5.

Additionally, Saraiva et al. developed miR-124 loaded NPs, which were able to deliver miR-124 into neural stem cells, and then promoted subventricular zone neurogenesis and maturation in PD [130]. More importantly, miR-124 NPs enhanced the migration of new neurons into 6-OHDA lesioned striatum, culminating in improved motor function and brain repair in a PD mouse model [130], confirming the effect of miR-124NPs as a therapeutic approach to boost endogenous brain repair in PD.

**Figure 3:**
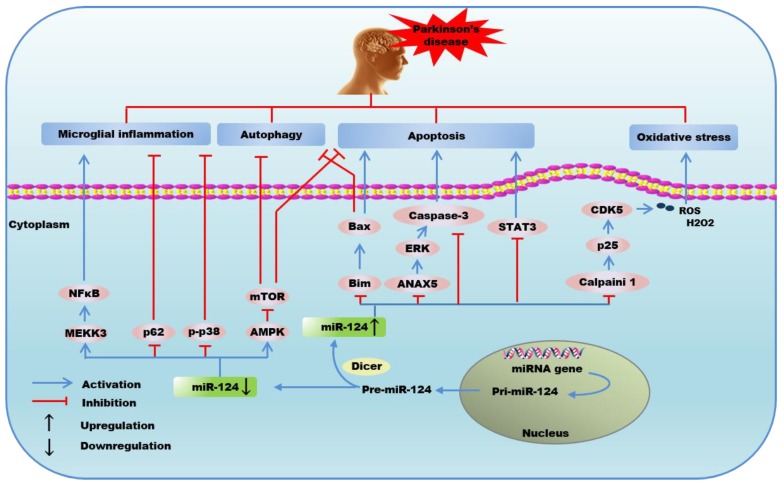
The possible mechanisms of the regulation of microRNA-124 in Parkinson’s disease.

In summary, miR-124 contributes to neuroprotective effects in PD via modulating apoptosis, autophagy, inflammation, oxidative stress, and neurogenesis. Present studies reveal that miR-124 attenuates neuronal apoptosis and autophagy in PD via downregulating Bim [23], AMPK [48], STAT3 [49], ANAX5 [50], and caspase3 [51]. Moreover, overexpression of miR-124 could effectively ameliorate microglial inflammatory response by targeting and inhibiting p62, p-p38 [53], and MEKK3/NF-kB signaling pathway [52]. In addition, it is clearly evident that miR-124 acts to diminish the expression of calpain 1/p25/cdk5 proteins in dopaminergic neurons, and thereby inhibits oxidative stress through scavenging ROS and H_2_O_2_ [17]. Furthermore, the administration of miR-124 NPs may function as a new therapeutic approach to enhance brain repair in PD by promoting subventricular zone neurogenesis [130] (Figure 3).

### 2.4. MiR-124 and Epilepsy

#### 2.4.1. Epilepsy 

Epilepsy is a chronic neurological disorder that is characterized by recurrent unprovoked seizures due to an imbalance of inhibitory and excitatory neurons in CNS [131]. Hyperexcitability and hypersynchrony of neuronal networks are the major pathogenies that contribute to the epileptic activity. A new theory has been put forward saying that genetic defects, as well as cerebral hypoperfusion are responsible for the negative consequences of epilepsy [132] One of the prominent pathological characteristics of ischemic stroke is reactive astrocytes, which then express higher glial fibrillary acidic protein and eventually trigger astroglial scar formation [133], which is the major effect on neuronal excitability [75,76]. Moreover, ischemia-induced BBB disruption, an anatomic alteration after cerebral ischemia [1], is also a fundamental catalyst for the initiation of epilepsy [134]. A population-based study of seizure revealed that cerebral infarction was a common cause of epilepsy [135,136], and patients with a disabling cortical infarct are more likely to have epilepsy after ischemic stroke [137]. Though the mechanisms underlying epileptogenesis remain uncertain, inflammation, mitochondrial dysfunction, and oxidative stress are emerging as the major factors contributing to epileptogenesis [138]. Furthermore, inflammatory response and synaptic reorganization of hippocampal structures are considered as potential mechanisms underlying hippocampal hyperexcitability in patients with epilepsy [139,140], while miR-124 has been found to inhibit hyperexcitability after seizure activity [32]. Moreover, the altered level of miR-124 has been confirmed in experimental and human epilepsy, which implies diagnostic effect and therapeutic potential of miR-124 in the pathogenesis of epilepsy or seizures [141].

#### 2.4.2. Mechanisms of miR-124 in Epilepsy 

A previous study has suggested that cAMP-response element (CRE) transcription is involved in the development of epilepsy and suppressing CREB activity might be a therapeutic strategy for decreasing the severity of epilepsy [142]. Moreover, Wang et al. reported that hippocampal pretreatment with miR-124 duplex gave rise to the alleviation of neuronal excitability and seizure severity, and prolonged onset latency via directly targeting 3’UTR of *CREB1* gene and inhibiting expression of CREB1 [32]. However, Brennan et al. demonstrated that miR-124 played a dual and opposing role in epileptogenesis. They found that miR-124 downregulated the level of C/EBPα, which then blocked the expression of neuron restrictive silencer factor (NRSF), a transcriptional suppressor that was identified as a contributor to epileptogenesis [54]. However, miR-124 also augmented microglia activation and inflammatory cytokines, including IL-6, IL-1β, and TNF-α in epileptogenesis as well [54]. Epilepsy has been found to result in cell death by the activation of caspase-3, which can mediate epilepsy-induced neurocytes apoptosis [143]. Schouten et al. reported that miR-124 and miR-137 both upregulated after kainic acid-induced seizures. Moreover, the two cooperative miRNAs mediated reduction of Bcl-2-like 13 (Bcl2L13), a novel target of miR-124 and miR-137, correlated with a significant reduction in caspase-3 activity, subsequently fine-tuning the mitochondrial-dependent apoptotic pathway in neural stem/progenitor cells exposed to kainic acid [55]. Therefore, miR-124 could trigger apoptosis-inhibitory pathway via targeting Bcl2L13. These studies underscore that miR-124 can be a potential target for anticonvulsant drugs in epilepsy, and provide new insight into the mechanisms underlying epilepsy as well.

**Figure 4:**
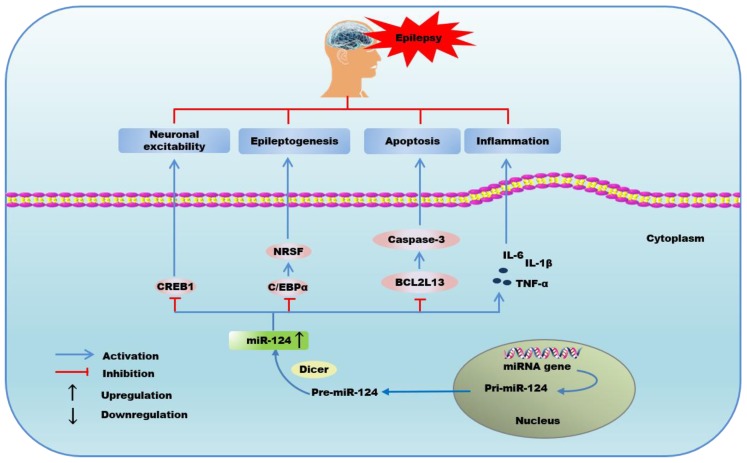
The possible mechanisms of the regulation of microRNA-124 in epilepsy or seizures.

In summary, miR-124 exerts a dual role in epilepsy or seizures. MiR-124 can attenuate epileptogenesis by inhibiting apoptosis, neuronal excitability, and prolonging onset latency via inhibiting expression of CREB1 [32], C/EBPα [54], and Bcl2L13 [55], while promoting epilepsy through microglia activation and inflammatory response [54] (Figure 4).

## 3. Conclusions

Cerebral ischemia injury, which results from insufficient blood flow, oxygen, and nutrients delivery to the brain, can initiate sequential molecular and cellular pathologies that underlie age-related IE, such as IS, AD, PD, epilepsy, etc. As a miRNA specifically expressed in the brain, miR-124 changes significantly in cerebral ischemia injury. Notably, miR-124 exerts paradoxical effects in a variety of physiological and pathological processes that participate in age-related IE and provides reason for further exploration. miR-124 plays a beneficial role in age-related IE via regulating autophagy, neuroinflammation, oxidative stress, neuronal excitability, neurodifferentiation, Aβ deposition, and hyperphosphorylation of tau protein, while playing a dual role via regulating apoptosis and causing detrimental effects on synaptic plasticity and axonal growth.

In conclusion, miR-124 cannot only exist as a potential diagnostic biomarker and indicator of the degree of brain damage in age-related IE, but also possesses the potential to function as a therapeutic target for the treatment of age-related IE and predictor of the prognosis of IS. Moreover, exosomes may function as novel therapeutic drug carriers and delivery vehicles across BBB. However, the multiplicity of binding targets poses potential difficulties, the modulation of miR-124 may open new avenues for therapeutic approaches to age-related IE. Further dissection of the pathophysiological mechanisms concerning miR-124 and age-related IE, and the further application of exosomal miR-124 may lead to new therapeutic opportunities for treating cerebral ischemia injury.

## Figures and Tables

**Table 1:** Possible targets of microRNA-124 in ischemic encephalopathy.

Diseases	Targets	Abbreviations	Function	Effects	Refs
**Ischemic stroke**	SRY-box transcription factor	Sox9	Neuronal differentiation	Repressing the expression of Sox9	[24]
	Jagged-1	JAG1	Neuronal differentiation	Repressing JAG1/Notch signaling pathway	[33]
	Ubiquitin specific peptidase 14	Usp14	Neuronal differentiation Neurovascular remodeling	Decreasing the expression of Usp14, which mediates REST degradation	[37]
			Anti-apoptosis	Exosomal miR-124 derived from M2 microglia attenuates neuronal apoptosis via downregulating Usp14	[38]
	Phosphoinositide 3-kinase	PI3K	Anti-axonal growth	Inhibition of miR-124 activating PI3K/AKT/mTOR/GAP-43 pathway	[39]
			Anti-apoptosis	Activating PI3K/AKT/ Bcl-2 signaling pathway	[40]
				Activating PI3K/AKT/Nrf2 signaling pathway	[41]
			Anti-oxidative stress	Activating PI3K/AKT/Nrf2 signaling pathway	[41]
	Inhibitory member of the apoptosis-stimulating proteins of p53 family	iASPP	Pro-apoptosis	Downregulating the expression of iASPP	[36]
	B-cell lymphoma-2 B-cell lymphoma-extra large	Bcl-2/Bcl-xl	Anti-apoptosis	Upregulating the expression of Bcl-2 and Bcl-xl respectively	[42]
	Janus kinase 2	*JAK2*	Anti-apoptosis	Activating JAK2/STAT3/Bcl-2 signaling pathway	[16]
	X-ray repair cross-complementing protein 6	Ku70	Pro-apoptosis	Knockdown of miR-124 attenuates I/R-induced apoptosis via negatively regulating Ku 70	[43]
	CCAAT/enhancer-binding protein alpha	C/EBP-α	Anti-inflammation	Inhibiting C/EBP-α/PU.1 signaling pathway	[22]
	RE1-silencing transcription factor	REST	Neuroplasticity Angiogenesis	Facilitating REST degradation	[44]
**Alzheimer’s disease**	Regulatory factor X1	RFX1	Anti-Aβ deposition	Decreased miR-124 represses *ApoE*-dependent Aβ uptake by targeting and upregulating RFX1	[34]
	cAMP-response element binding protein	CREB	Suppressing synaptic plasticity	Decreased miR-124 increases the expression of CREB and subsequent BDNF synthesis	[45]
	Tyrosine-protein phosphatase non-receptor type 1	PTPN1	Suppressing synaptic plasticity	Downregulating the expression of PTPN1	[46]
	Beta-site Amyloid precursor protein Cleaving Enzyme 1	BACE1	Anti- Aβ deposition	Decreased miR-124 upregulates BACE1 level	[47]
	Caveolin-1	Cav-1	Inhibiting hyperphosphorylation of tau	Repressing the expression of Cav-1, which further activates PI3K/AKT/GSK3β signaling pathway	[35]
**Parkinson’s disease**	Bim	Bim	Anti-apoptosis Anti-autophagy	Inhibiting the expression of Bim and reducing Bax translocation to mitochondria and lysosomal membrane	[23]
	Adenosine 5’-monophosphate-activated protein kinase	AMPK	Anti-apoptosis Anti-autophagy	Decreased miR-124 induces apoptosis and autophagy-associated proteins via activating AMPK/mTOR signaling pathway	[48]
	Signal transducer and activator of transcription-3	STAT3	Anti-apoptosis	Suppressing STAT3 expression	[49]
	AnnexinA5	ANXA5	Anti-apoptosis	Downregulating ANXA5/ERK signaling pathway	[50]
	Caspase-3	Casp-3	Anti-apoptosis	Restraining the increase of Casp-3	[51]
	Mitogen-activated protein kinase kinase kinase 3	MEKK3	Anti-neuroinflammation	Suppressing MEKK3/NF-κB signaling pathway	[52]
	Sequestosome 1 Phospho-p38 mitogen-activated protein kinase	p62 p-p38	Anti-inflammation	Suppressing the expression of p62 and p-p38	[53]
	Calpain 1	Calpain 1	Anti-oxidative stress	Decreased miR-124 upregulates the expression of calpain1/p25/CDK5 pathway proteins	[17]
**Epilepsy**	cAMP-response element-binding protein 1	CREB1	Anti-neuronal excitability	Repressing the expression of CREB1	[32]
	CCAAT/enhancer-binding protein alpha	C/EBPα	Anti-epileptogenic	Downregulating C/EBPα level, which then inhibits the expression and activity of NRSF	[54]
	Bcl-2-like 13	Bcl2L13	Anti-apoptosis	Diminishing the expression of Bcl2L13, which further represses the activation of caspase-3	[55]

## Abbreviations

IE	Ischemic encephalopathy
AD	Alzheimer’s disease
PD	Parkinson’s disease
MiR-124	MicroRNA-124
BBB	Blood-brain barrier
MiRNAs	MicroRNAs
CNS	Central nervous system
CIRI	Cerebral ischemia reperfusion injury
VSNL-1	Visinin-like protein-1
CDK4	Cyclin-dependent kinase family 4
Sox9	SRY-box transcription factor
OGD	Oxygen and glucose deprivation
JAG1	Jagged-1
REST	RE1-silencing transcription factor
Usp14	Ubiquitin specific peptidase 14
PI3K	Phosphoinositide 3-kinase
mTOR	Mammalian target of rapamycin
PKB/AKT	Protein kinase B
GAP-43	Growth associated protein-43
TNF-α	Necrosis factor-α
IL-10	Interleukin -10
C/EBP-α	CCAAT/enhancer-binding protein alpha
Bcl-2	B-cell Lymphoma-2
iASPP	Inhibitory member of the apoptosis-stimulating proteins of p53 family
Bcl-xl	B-cell lymphoma-extra large
I/R	Ischemia/reperfusion
JAK2	Janus tyrosine kinase 2
STAT3	Signal transducer and activator of transcription 3
Ku70	X-ray repair cross-complementing protein 6
GLT1	Glutamate transporter 1
Aβ	β-amyloid
NFTs	Neurofibrillary tangles
BACE1	Beta-site amyloid precursor protein cleaving enzyme1
GSK3β	Glycogen synthase kinase 3β
RFX1	Regulatory factor X1
ApoE	Apolipoprotein E
CREB	cAMP-response element-binding protein
BDNF	Brain-derived neurotrophic factor
PTPN1	Tyrosine-protein phosphatase non-receptor type 1
SNpc	Substantia nigra pars compacta
MPTP	1-methyl-4-pheny-1,2,3,6-tetrahydropyridine
MPP	Methyl phenyl pyridinium
AMPK	Adenosine 5’-monophosphate-activated protein kinase
ANAX5	Annexin 5
ERK	Extracellular regulated protein kinases
Casp-3	Caspase-3
MALAT1	Metastasis-associated lung adenocarcinoma transcript 1
MEKK3	Mitogen-activated protein kinase kinase kinase 3
p62	Sequestosome 1
p-p38	Phosphor-p38 mitogen-activated protein kinase
CRE	cAMP-response element
NRSF	Neuron restrictive silencer factor

## References

[1] Chen Z.X., Xu Q.Q., Shan C.S., Shi Y.H., Wang Y., Chang R.C., Zheng G.Q. (2019). Borneol for Regulating the Permeability of the Blood-Brain Barrier in Experimental Ischemic Stroke: Preclinical Evidence and Possible Mechanism. Oxid. Med. Cell Longev..

[2] Rodrigo R., Fernandez-Gajardo R., Gutierrez R., Matamala J.M., Carrasco R., Miranda-Merchak A., Feuerhake W. (2013). Oxidative stress and pathophysiology of ischemic stroke: Novel therapeutic opportunities. CNS Neurol. Disord. Drug Targets.

[3] Khoshnam S.E., Winlow W., Farzaneh M., Farbood Y., Moghaddam H.F. (2017). Pathogenic mechanisms following ischemic stroke. Neurol. Sci..

[4] Graf R., Kataoka K., Rosner G., Heiss W.D. (1986). Cortical deafferentation in cat focal ischemia: Disturbance and recovery of sensory functions in cortical areas with different degrees of cerebral blood flow reduction. J. Cereb. Blood Flow Metab..

[5] Sun F., Wang X., Mao X., Xie L., Jin K. (2012). Ablation of neurogenesis attenuates recovery of motor function after focal cerebral ischemia in middle-aged mice. PLoS ONE.

[6] Escobar I., Xu J., Jackson C.W., Perez-Pinzon M.A. (2019). Altered Neural Networks in the Papez Circuit: Implications for Cognitive Dysfunction after Cerebral Ischemia. J. Alzheimers Dis..

[7] Graham S.H., Liu H. (2017). Life and death in the trash heap: The ubiquitin proteasome pathway and UCHL1 in brain aging, neurodegenerative disease and cerebral Ischemia. Ageing Res. Rev..

[8] Sommer J.C. (2017). Ischemic stroke: experimental models and reality. Acta Neuropathol..

[9] Li J., Zhang M., Xu Z.Q., Gao C.Y., Fang C.Q., Deng J., Yan J.C., Wang Y.J., Zhou H.D. (2010). Vascular risk aggravates the progression of Alzheimer’s disease in a Chinese cohort. J Alzheimers Dis..

[10] Kim T., Vemuganti R. (2017). Mechanisms of Parkinson’s disease-related proteins in mediating secondary brain damage after cerebral ischemia. J Cereb. Blood Flow Metab..

[11] Jaenisch N., Liebmann L., Guenther M., Hubner C.A., Frahm C., Witte O.W. (2016). Reduced tonic inhibition after stroke promotes motor performance and epileptic seizures. Sci. Rep..

[12] Radak D., Katsiki N., Resanovic I., Jovanovic A., Sudar-Milovanovic E., Zafirovic S., Mousad S.A., Isenovic E.R. (2017). Apoptosis and Acute Brain Ischemia in Ischemic Stroke. Curr. Vasc. Pharmacol..

[13] Hamzei T.S., Kho W., Aswendt M., Collmann F.M., Green C., Adamczak J., Tennstaedt A., Hoehn M. (2016). Dynamic Modulation of Microglia/Macrophage Polarization by miR-124 after Focal Cerebral Ischemia. J. Neuroimmune Pharmacol..

[14] Ozkul A., Akyol A., Yenisey C., Arpaci E., Kiylioglu N., Tataroglu C. (2007). Oxidative stress in acute ischemic stroke. J. Clin. Neurosci..

[15] Zhao F., Qu Y., Zhu J., Zhang L., Huang L., Liu H., Li S., Mu D. (2017). MiR-30d-5p Plays an Important Role in Autophagy and Apoptosis in Developing Rat Brains After Hypoxic-Ischemic Injury. J. Neuropathol. Exp. Neurol..

[16] Zhang W., Meng A. (2019). MicroRNA-124 expression in the brains of rats during early cerebral ischemia and reperfusion injury is associated with cell apoptosis involving STAT3. Exp. Ther. Med..

[17] Kanagaraj N., Beiping H., Dheen S.T., Tay S.S. (2014). Downregulation of miR-124 in MPTP-treated mouse model of Parkinson’s disease and MPP iodide-treated MN9D cells modulates the expression of the calpain/cdk5 pathway proteins. Neuroscience.

[18] Lewis B.P., Burge C.B., Bartel D.P. (2005). Conserved seed pairing, often flanked by adenosines, indicates that thousands of human genes are microRNA targets. Cell.

[19] Khoshnam S.E., Winlow W., Farbood Y., Moghaddam H.F., Farzaneh M. (2017). Emerging Roles of microRNAs in Ischemic Stroke: As Possible Therapeutic Agents. J. Stroke.

[20] Deo M., Yu J.Y., Chung K.H., Tippens M., Turner D.L. (2006). Detection of mammalian microRNA expression by in situ hybridization with RNA oligonucleotides. Dev. Dyn..

[21] Akerblom M., Sachdeva R., Barde I., Verp S., Gentner B., Trono D., Jakobsson J. (2012). MicroRNA-124 is a subventricular zone neuronal fate determinant. J. Neurosci..

[22] Ponomarev E.D., Veremeyko T., Barteneva N., Krichevsky A.M., Weiner H.L. (2011). MicroRNA-124 promotes microglia quiescence and suppresses EAE by deactivating macrophages via the C/EBP-alpha-PU.1 pathway. Nat. Med..

[23] Wang H., Ye Y., Zhu Z., Mo L., Lin C., Wang Q., Wang H., Gong X., He X., Lu G. (2016). MiR-124 Regulates Apoptosis and Autophagy Process in MPTP Model of Parkinson’s Disease by Targeting to Bim. Brain Pathol..

[24] Cheng L.C., Pastrana E., Tavazoie M., Doetsch F. (2009). MiR-124 regulates adult neurogenesis in the subventricular zone stem cell niche. Nat. Neurosci..

[25] Morel L., Regan M., Higashimori H., Ng S.K., Esau C., Vidensky S., Rothstein J., Yang Y. (2013). Neuronal exosomal miRNA-dependent translational regulation of astroglial glutamate transporter GLT1. J. Biol. Chem..

[26] Hamzei T.S., Kho W., Riou A., Wiedermann D., Hoehn M. (2016). MiRNA-124 induces neuroprotection and functional improvement after focal cerebral ischemia. Biomaterials.

[27] Rajasethupathy P., Fiumara F., Sheridan R., Betel D., Puthanveettil S.V., Russo J.J., Sander C., Tuschl T., Kandel E. (2009). Characterization of small RNAs in Aplysia reveals a role for miR-124 in constraining synaptic plasticity through CREB. Neuron.

[28] Kulkarni V.A., Firestein B.L. (2012). The dendritic tree and brain disorders. Mol. Cell Neurosci..

[29] Sonntag K.C., Woo T.U., Krichevsky A.M. (2012). Converging miRNA functions in diverse brain disorders: A case for miR-124 and miR-126. Exp. Neurol..

[30] Soreq H., Wolf Y. (2011). NeurimmiRs: MicroRNAs in the neuroimmune interface. Trends Mol. Med..

[31] Volny O., Kasickova L., Coufalova D., Cimflova P., Novak J. (2015). MicroRNAs in Cerebrovascular Disease. Adv. Exp. Med. Biol..

[32] Wang W., Wang X., Chen L., Zhang Y., Xu Z., Liu J., Jiang G., Li J., Zhang X., Wang K. (2016). The microRNA miR-124 suppresses seizure activity and regulates CREB1 activity. Expert Rev. Mol. Med..

[33] Liu X.S., Chopp M., Zhang R.L., Tao T., Wang X.L., Kassis H., Hozeska-Solgot A., Zhang L., Chen C., Zhang Z.G. (2011). MicroRNA profiling in subventricular zone after stroke: MiR-124a regulates proliferation of neural progenitor cells through Notch signaling pathway. PLoS ONE.

[34] Feng C.Z., Yin J.B., Yang J.J., Cao L. (2017). Regulatory factor X1 depresses ApoE-dependent Abeta uptake by miRNA-124 in microglial response to oxidative stress. Neuroscience.

[35] Kang Q., Xiang Y., Li D., Liang J., Zhang X., Zhou F., Qiao M., Nie Y., He Y., Cheng J. (2017). MiR-124-3p attenuates hyperphosphorylation of Tau protein-induced apoptosis via caveolin-1-PI3K/Akt/GSK3beta pathway in N2a/APP695swe cells. Oncotarget.

[36] Liu X., Li F., Zhao S., Luo Y., Kang J., Zhao H., Yan F., Li S., Ji X. (2013). MicroRNA-124-mediated regulation of inhibitory member of apoptosis-stimulating protein of p53 family in experimental stroke. Stroke.

[37] Doeppner T.R., Doehring M., Bretschneider E., Zechariah A., Kaltwasser B., Muller B., Koch J.C., Bahr M., Hermann D.M., Michel U. (2013). MicroRNA-124 protects against focal cerebral ischemia via mechanisms involving Usp14-dependent REST degradation. Acta Neuropathol..

[38] Song Y., Li Z., He T., Qu M., Jiang L., Li W., Shi X., Pa J., Zhang L., Wang Y. (2019). M2 microglia-derived exosomes protect the mouse brain from ischemia-reperfusion injury via exosomal miR-124. Theranostics.

[39] Zhu H., Wang J., Shao Y., Wan D. (2019). Catalpol may improve axonal growth via regulating miR-124 regulated PI3K/AKT/mTOR pathway in neurons after ischemia. Ann. Transl. Med..

[40] Wang C., Wei Z., Jiang G., Liu H. (2017). Neuroprotective mechanisms of miR-124 activating PI3K/Akt signaling pathway in ischemic stroke. Exp. Ther. Med..

[41] Shu K., Zhang Y. (2019). Protodioscin protects PC12 cells against oxygen and glucose deprivation-induced injury through miR-124/AKT/Nrf2 pathway. Cell Stress Chaperones.

[42] Sun Y., Gui H., Li Q., Luo Z.M., Zheng M.J., Duan J.L., Liu X. (2013). MicroRNA-124 protects neurons against apoptosis in cerebral ischemic stroke. CNS Neurosci. Ther..

[43] Zhu F., Liu J.L., Li J.P., Xiao F., Zhang Z.X., Zhang L. (2014). MicroRNA-124 (miR-124) regulates Ku70 expression and is correlated with neuronal death induced by ischemia/reperfusion. J. Mol. Neurosci..

[44] Doeppner T.R., Kaltwasser B., Sanchez-Mendoza E.H., Caglayan A.B., Bahr M., Hermann D.M. (2017). Lithium-induced neuroprotection in stroke involves increased miR-124 expression, reduced RE1-silencing transcription factor abundance and decreased protein deubiquitination by GSK3beta inhibition-independent pathways. J. Cereb. Blood Flow Metab..

[45] Zhao Y.N., Li W.F., Li F., Zhang Z., Dai Y.D., Xu A.L., Qi C., Gao J.M., Gao J. (2013). Resveratrol improves learning and memory in normally aged mice through microRNA-CREB pathway. Biochem. Biophys. Res. Commun..

[46] Wang X., Liu D., Huang H.Z., Wang Z.H., Hou T.Y., Yang X., Pang P., Wei N., Zhou Y.F., Dupras M.J. (2018). A Novel MicroRNA-124/PTPN1 Signal Pathway Mediates Synaptic and Memory Deficits in Alzheimer’s Disease. Biol. Psychiatr..

[47] An F., Gong G., Wang Y., Bian M., Yu L., Wei C. (2017). MiR-124 acts as a target for Alzheimer’s disease by regulating BACE1. Oncotarget.

[48] Gong X., Wang H., Ye Y., Shu Y., Deng Y., He X., Lu G., Zhang S. (2016). MiR-124 regulates cell apoptosis and autophagy in dopaminergic neurons and protects them by regulating AMPK/mTOR pathway in Parkinson’s disease. Am. J. Transl. Res..

[49] Geng L., Liu W., Chen Y. (2017). MiR-124-3p attenuates MPP (+)-induced neuronal injury by targeting STAT3 in SH-SY5Y cells. Exp. Biol. Med..

[50] Dong R.F., Zhang B., Tai L.W., Liu H.M., Shi F.K., Liu N.N. (2018). The Neuroprotective Role of MiR-124-3p in a 6-Hydroxydopamine-Induced Cell Model of Parkinson’s Disease via the Regulation of ANAX5. J. Cell Biochem..

[51] Liu W., Zhang Q., Zhang J., Pan W., Zhao J., Xu Y. (2017). Long non-coding RNA MALAT1 contributes to cell apoptosis by sponging miR-124 in Parkinson disease. Cell Biosci..

[52] Yao L., Ye Y., Mao H., Lu F., He X., Lu G., Zhang S. (2018). MicroRNA-124 regulates the expression of MEKK3 in the inflammatory pathogenesis of Parkinson’s disease. J. Neuroinflamm..

[53] Yao L., Zhu Z., Wu J., Zhang Y., Zhang H., Sun X., Qian C., Wang B., Xie L., Zhang S. (2019). MicroRNA-124 regulates the expression of p62/p38 and promotes autophagy in the inflammatory pathogenesis of Parkinson’s disease. FASEB J..

[54] Brennan G.P., Dey D., Chen Y., Patterson K.P., Magnetta E.J., Hall A.M., Dube C.M., Mei Y.T., Baram T.Z. (2016). Dual and Opposing Roles of MicroRNA-124 in Epilepsy Are Mediated through Inflammatory and NRSF-Dependent Gene Networks. Cell Rep..

[55] Schouten M., Fratantoni S.A., Hubens C.J., Piersma S.R., Pham T.V., Bielefeld P., Voskuyl R.A., Lucassen P.J., Jimenez C.R., Fitzsimons C.P. (2015). MicroRNA-124 and -137 cooperativity controls caspase-3 activity through BCL2L13 in hippocampal neural stem cells. Sci. Rep..

[56] Feigin V.L., Roth G.A., Naghavi M., Parmar P., Krishnamurthi R., Chugh S., Mensah G.A., Norrving B., Shiue I., Ng M. (2016). Global burden of stroke and risk factors in 188 countries, during 1990–2013: A systematic analysis for the Global Burden of Disease Study 2013. Lancet Neurol..

[57] Toni D., Fiorelli M., Gentile M., Bastianello S., Sacchetti M.L., Argentino C., Pozzilli C., Fieschi C. (1995). Progressing neurological deficit secondary to acute ischemic stroke. A study on predictability, pathogenesis, and prognosis. Arch. Neurol..

[58] Zheng Z., Zhao H., Steinberg G.K., Yenari M.A. (2003). Cellular and molecular events underlying ischemia-induced neuronal apoptosis. Drug News Perspect..

[59] Felling R.J., Song H. (2015). Epigenetic mechanisms of neuroplasticity and the implications for stroke recovery. Exp. Neurol..

[60] Sun M., Hou X., Ren G., Zhang Y., Cheng H. (2019). Dynamic changes in miR-124 levels in patients with acute cerebral infarction. Int. J. Neurosci..

[61] Chen S.H., Sun H., Zhang Y.M., Xu H., Yang Y., Wang F.M. (2016). Effects of acupuncture at Baihui (GV 20) and Zusanli (ST 36) on peripheral serum expression of MicroRNA 124, laminin and integrin beta1 in rats with cerebral ischemia reperfusion injury. Chin. J. Integr. Med..

[62] Weng H., Shen C., Hirokawa G., Ji X., Takahashi R., Shimada K., Kishimoto C., Iwai N. (2011). Plasma miR-124 as a biomarker for cerebral infarction. Biomed. Res..

[63] Ji Q., Ji Y., Peng J., Zhou X., Chen X., Zhao H., Xu T., Chen L., Xu Y. (2016). Increased Brain-Specific MiR-9 and MiR-124 in the Serum Exosomes of Acute Ischemic Stroke Patients. PLoS ONE.

[64] Rainer T.H., Leung L.Y., Chan C., Leung Y.K., Abrigo J.M., Wang D., Graham C.A. (2016). Plasma miR-124-3p and miR-16 concentrations as prognostic markers in acute stroke. Clin. Biochem..

[65] Jeyaseelan K., Lim K.Y., Armugam A. (2008). MicroRNA expression in the blood and brain of rats subjected to transient focal ischemia by middle cerebral artery occlusion. Stroke.

[66] Chen S., Chen Y., Gao Y., Zuo Y., Zhou X. (2018). Effect of single-nucleotide polymorphism in pri-microRNA-124 on poststroke motor function recovery. J. Cell Biochem..

[67] Saraiva C., Talhada D., Rai A., Ferreira R., Ferreira L., Bernardino L., Ruscher K. (2018). MicroRNA-124-loaded nanoparticles increase survival and neuronal differentiation of neural stem cells in vitro but do not contribute to stroke outcome in vivo. PLoS ONE.

[68] Yu Y.L., Chou R.H., Shyu W.C., Hsieh S.C., Wu C.S., Chiang S.Y., Chang W.J., Chen J.N., Tseng Y.J., Lin Y.H. (2013). Smurf2-mediated degradation of EZH2 enhances neuron differentiation and improves functional recovery after ischaemic stroke. EMBO Mol. Med..

[69] Wang L., Chopp M., Zhang R.L., Zhang L., Letourneau Y., Feng Y.F., Jiang A., Morris D.C., Zhang Z.G. (2009). The Notch pathway mediates expansion of a progenitor pool and neuronal differentiation in adult neural progenitor cells after stroke. Neuroscience.

[70] Lee Y., Lee S.R., Choi S.S., Yeo H.G., Chang K.T., Lee H.J. (2014). Therapeutically targeting neuroinflammation and microglia after acute ischemic stroke. Biomed. Res. Int..

[71] Hu X., Li P., Guo Y., Wang H., Leak R.K., Chen S., Gao Y., Chen J. (2012). Microglia/macrophage polarization dynamics reveal novel mechanism of injury expansion after focal cerebral ischemia. Stroke.

[72] Ning R., Venkat P., Chopp M., Zacharek A., Yan T., Cui X., Seyfried D., Chen J. (2017). D-4F increases microRNA-124a and reduces neuroinflammation in diabetic stroke rats. Oncotarget.

[73] Ponomarev E.D., Veremeyko T., Weiner H.L. (2013). MicroRNAs are universal regulators of differentiation, activation, and polarization of microglia and macrophages in normal and diseased CNS. Glia.

[74] Xu L., He D., Bai Y. (2016). Microglia-Mediated Inflammation and Neurodegenerative Disease. Mol. Neurobiol..

[75] Pivonkova H., Anderova M. (2017). Altered Homeostatic Functions in Reactive Astrocytes and Their Potential as a Therapeutic Target After Brain Ischemic Injury. Curr. Pharm. Des..

[76] Hamill C.E., Goldshmidt A., Nicole O., McKeon R.J., Brat D.J., Traynelis S.F. (2005). Special lecture: Glial reactivity after damage: Implications for scar formation and neuronal recovery. Clin. Neurosurg..

[77] Hu G.Q., Du X., Li Y.J., Gao X.Q., Chen B.Q., Yu L. (2017). Inhibition of cerebral ischemia/reperfusion injury-induced apoptosis: Nicotiflorin and JAK2/STAT3 pathway. Neural Regen. Res..

[78] Sawada M., Sun W., Hayes P., Leskov K., Boothman D.A., Matsuyama S. (2003). Ku70 suppresses the apoptotic translocation of Bax to mitochondria. Nat. Cell Biol..

[79] Aryani A., Denecke B. (2016). Exosomes as a Nanodelivery System: A Key to the Future of Neuromedicine?. Mol. Neurobiol..

[80] Valadi H., Ekstrom K., Bossios A., Sjostrand M., Lee J.J., Lotvall J.O. (2007). Exosome-mediated transfer of mRNAs and microRNAs is a novel mechanism of genetic exchange between cells. Nat. Cell Biol..

[81] Zhang Z.G., Chopp M. (2016). Exosomes in stroke pathogenesis and therapy. J. Clin. Investig..

[82] Lee S.T., Im W., Ban J.J., Lee M., Jung K.H., Lee S.K., Chu K., Kim M. (2017). Exosome-Based Delivery of miR-124 in a Huntington’s Disease Model. J. Mov. Disord..

[83] Yang J., Zhang X., Chen X., Wang L., Yang G. (2017). Exosome Mediated Delivery of miR-124 Promotes Neurogenesis after Ischemia. Mol. Ther. Nucleic Acids.

[84] Mesulam M.M. (1999). Neuroplasticity failure in Alzheimer’s disease: Bridging the gap between plaques and tangles. Neuron.

[85] Alzheimer’s Association (2015). 2015 Alzheimer’s disease facts and figures. Alzheimers Dement..

[86] Hyman B.T., Phelps C.H., Beach T.G., Bigio E.H., Cairns N.J., Carrillo M.C., Dickson D.W., Duyckaerts C., Frosch M.P., Masliah E. (2012). National Institute on Aging-Alzheimer’s Association guidelines for the neuropathologic assessment of Alzheimer’s disease. Alzheimers Dement..

[87] Guglielmotto M., Aragno M., Autelli R., Giliberto L., Novo E., Colombatto S., Danni O., Parola M., Smith M.A., Perry G. (2009). The up-regulation of BACE1 mediated by hypoxia and ischemic injury: Role of oxidative stress and HIF1alpha. J. Neurochem..

[88] Cai Z., Liu Z., Xiao M., Wang C., Tian F. (2017). Chronic Cerebral Hypoperfusion Promotes Amyloid-Beta Pathogenesis via Activating beta/gamma-Secretases. Neurochem. Res..

[89] Zhao Y., Gu J.H., Dai C.L., Liu Q., Iqbal K., Liu F., Gong C.X. (2014). Chronic cerebral hypoperfusion causes decrease of O-GlcNAcylation, hyperphosphorylation of tau and behavioral deficits in mice. Front. Aging Neurosci..

[90] Pluta R., Jablonski M., Ulamek-Koziol M., Kocki J., Brzozowska J., Januszewski S., Furmaga-Jablonska W., Bogucka-Kocka A., Maciejewski R., Czuczwar S.J. (2013). Sporadic Alzheimer’s disease begins as episodes of brain ischemia and ischemically dysregulated Alzheimer’s disease genes. Mol. Neurobiol..

[91] Salminen A., Kauppinen A., Kaarniranta K. (2017). Hypoxia/ischemia activate processing of Amyloid Precursor Protein: Impact of vascular dysfunction in the pathogenesis of Alzheimer’s disease. J. Neurochem..

[92] Hampel H., Shen Y. (2009). Beta-site amyloid precursor protein cleaving enzyme 1 (BACE1) as a biological candidate marker of Alzheimer’s disease. Scand. J. Clin. Lab. Invest..

[93] Fang M., Wang J., Zhang X., Geng Y., Hu Z., Rudd J.A., Ling S., Chen W., Han S. (2012). The miR-124 regulates the expression of BACE1/beta-secretase correlated with cell death in Alzheimer’s disease. Toxicol. Lett..

[94] Irie K., Murakami K., Masuda Y., Morimoto A., Ohigashi H., Ohashi R., Takegoshi K., Nagao M., Shimizu T., Shirasawa T. (2005). Structure of beta-amyloid fibrils and its relevance to their neurotoxicity: Implications for the pathogenesis of Alzheimer’s disease. J. Biosci. Bioeng..

[95] Rowan M.J., Klyubin I., Cullen W.K., Anwyl R. (2003). Synaptic plasticity in animal models of early Alzheimer’s disease. Philos. Trans. R. Soc. Lond. B Biol. Sci..

[96] Zuroff L., Daley D., Black K.L., Koronyo-Hamaoui M. (2017). Clearance of cerebral Abeta in Alzheimer’s disease: Reassessing the role of microglia and monocytes. Cell Mol. Life Sci..

[97] Lee C.Y., Tse W., Smith J.D., Landreth G.E. (2012). Apolipoprotein E promotes beta-amyloid trafficking and degradation by modulating microglial cholesterol levels. J. Biol. Chem..

[98] Pugazhenthi S., Wang M., Pham S., Sze C.I., Eckman C.B. (2011). Downregulation of CREB expression in Alzheimer’s brain and in Abeta-treated rat hippocampal neurons. Mol. Neurodegener..

[99] Phillips H.S., Hains J.M., Armanini M., Laramee G.R., Johnson S.A., Winslow J.W. (1991). BDNF mRNA is decreased in the hippocampus of individuals with Alzheimer’s disease. Neuron.

[100] Deisseroth K., Bito H., Tsien R.W. (1996). Signaling from synapse to nucleus: Postsynaptic CREB phosphorylation during multiple forms of hippocampal synaptic plasticity. Neuron.

[101] Finkbeiner S., Tavazoie S.F., Maloratsky A., Jacobs K.M., Harris K.M., Greenberg M.E. (1997). CREB: A major mediator of neuronal neurotrophin responses. Neuron.

[102] Benito E., Barco A. (2010). CREB’s control of intrinsic and synaptic plasticity: Implications for CREB-dependent memory models. Trends Neurosci..

[103] Hwang K.D., Bak M.S., Kim S.J., Rhee S., Lee Y.S. (2017). Restoring synaptic plasticity and memory in mouse models of Alzheimer’s disease by PKR inhibition. Mol. Brain.

[104] Preethi J., Singh H.K., Charles P.D., Rajan K.E. (2012). Participation of microRNA 124-CREB pathway: A parallel memory enhancing mechanism of standardised extract of Bacopa monniera (BESEB CDRI-08). Neurochem. Res..

[105] Arancibia S., Silhol M., Mouliere F., Meffre J., Hollinger I., Maurice T., Tapia-Arancibia L. (2008). Protective effect of BDNF against beta-amyloid induced neurotoxicity in vitro and in vivo in rats. Neurobiol. Dis..

[106] Choi J., Kwon H.J., Lee J.E., Lee Y., Seoh J.Y., Han P.L. (2019). Hyperoxygenation revitalizes Alzheimer’s disease pathology through the upregulation of neurotrophic factors. Aging Cell.

[107] Chandrasekar V., Dreyer J.L. (2009). MicroRNAs miR-124, let-7d and miR-181a regulate cocaine-induced plasticity. Mol. Cell Neurosci..

[108] Zhao M.Y., Wang G.Q., Wang N.N., Yu Q.Y., Liu R.L., Shi W.Q. (2019). The long-non-coding RNA NEAT1 is a novel target for Alzheimer’s disease progression via miR-124/BACE1 axis. Neurol. Res..

[109] Hernandez F., Gomez D.B.E., Fuster-Matanzo A., Lucas J.J., Avila J. (2010). GSK3: A possible link between beta amyloid peptide and tau protein. Exp. Neurol..

[110] Huber C.M., Yee C., May T., Dhanala A., Mitchell C.S. (2018). Cognitive Decline in Preclinical Alzheimer’s Disease: Amyloid-Beta versus Tauopathy. J. Alzheimers Dis..

[111] Huang W., Cheng P., Yu K., Han Y., Song M., Li Y. (2017). Hyperforin attenuates aluminum-induced Abeta production and Tau phosphorylation via regulating Akt/GSK-3beta signaling pathway in PC12 cells. Biomed. Pharmacother..

[112] ArunSundar M., Shanmugarajan T.S., Ravichandiran V. (2018). 3,4-Dihydroxyphenylethanol Assuages Cognitive Impulsivity in Alzheimer’s Disease by Attuning HPA-Axis via Differential Crosstalk of alpha7 nAChR with MicroRNA-124 and HDAC6. ACS Chem. Neurosci..

[113] Beitz J.M. (2014). Parkinson’s disease: A review. Front. Biosci..

[114] Taylor J.M., Main B.S., Crack P.J. (2013). Neuroinflammation and oxidative stress: Co-conspirators in the pathology of Parkinson’s disease. Neurochem. Int..

[115] Dickson D.W. (2018). Neuropathology of Parkinson disease. Parkinsonism Relat. Disord..

[116] Alexander G.E. (2004). Biology of Parkinson’s disease: Pathogenesis and pathophysiology of a multisystem neurodegenerative disorder. Dialogues Clin. Neurosci..

[117] Tang H., Gao Y., Zhang Q., Nie K., Zhu R., Gao L., Feng S., Wang L., Zhao J., Huang Z. (2017). Chronic cerebral hypoperfusion independently exacerbates cognitive impairment within the pathopoiesis of Parkinson’s disease via microvascular pathologys. Behav. Brain Res..

[118] Rodriguez-Grande B., Blackabey V., Gittens B., Pinteaux E., Denes A. (2013). Loss of substance P and inflammation precede delayed neurodegeneration in the substantia nigra after cerebral ischemia. Brain Behav. Immun..

[119] Unal-Cevik I., Gursoy-Ozdemir Y., Yemisci M., Lule S., Gurer G., Can A., Muller V., Kahle P.J., Dalkara T. (2011). Alpha-synuclein aggregation induced by brief ischemia negatively impacts neuronal survival in vivo: A study in [A30P]alpha-synuclein transgenic mouse. J. Cereb. Blood Flow Metab..

[120] Burre J., Sharma M., Sudhof T.C. (2015). Definition of a molecular pathway mediating alpha-synuclein neurotoxicity. J. Neurosci..

[121] Li N., Pan X., Zhang J., Ma A., Yang S., Ma J., Xie A. (2017). Plasma levels of miR-137 and miR-124 are associated with Parkinson’s disease but not with Parkinson’s disease with depression. Neurol. Sci..

[122] Anglade P., Vyas S., Javoy-Agid F., Herrero M.T., Michel P.P., Marquez J., Mouatt-Prigent A., Ruberg M., Hirsch E.C., Agid Y. (1997). Apoptosis and autophagy in nigral neurons of patients with Parkinson’s disease. Histol. Histopathol..

[123] Vila M., Jackson-Lewis V., Vukosavic S., Djaldetti R., Liberatore G., Offen D., Korsmeyer S.J., Przedborski S. (2001). Bax ablation prevents dopaminergic neurodegeneration in the 1-methyl- 4-phenyl-1,2,3,6-tetrahydropyridine mouse model of Parkinson’s disease. Proc. Natl. Acad. Sci. USA.

[124] Xu Y., Liu C., Chen S., Ye Y., Guo M., Ren Q., Liu L., Zhang H., Xu C., Zhou Q. (2014). Activation of AMPK and inactivation of Akt result in suppression of mTOR-mediated S6K1 and 4E-BP1 pathways leading to neuronal cell death in in vitro models of Parkinson’s disease. Cell Signal..

[125] Czapski G.A., Gassowska M., Wilkaniec A., Cieslik M., Adamczyk A. (2013). Extracellular alpha-synuclein induces calpain-dependent overactivation of cyclin-dependent kinase 5 in vitro. FEBS Lett..

[126] Zhang P., Shao X.Y., Qi G.J., Chen Q., Bu L.L., Chen L.J., Shi J., Ming J., Tian B. (2016). Cdk5-Dependent Activation of Neuronal Inflammasomes in Parkinson’s Disease. Mov. Disord..

[127] Kanagaraj N. (2013). MicroRNA Expressions in the MPTP-Induced Parkinson’s Disease Model with Special Reference to miR-124 (Doctoral dissertation). https://core.ac.uk/download/pdf/48682603.pdf.

[128] Schirinzi T., Madeo G., Martella G., Maltese M., Picconi B., Calabresi P., Pisani A. (2016). Early synaptic dysfunction in Parkinson’s disease: Insights from animal models. Mov. Disord..

[129] Gutierrez-Vargas J.A., Munera A., Cardona-Gomez G.P. (2015). CDK5 knockdown prevents hippocampal degeneration and cognitive dysfunction produced by cerebral ischemia. J. Cereb. Blood Flow Metab..

[130] Saraiva C., Paiva J., Santos T., Ferreira L., Bernardino L. (2016). MicroRNA-124 loaded nanoparticles enhance brain repair in Parkinson’s disease. J. Control Release.

[131] Azam F., Prasad M.V., Thangavel N. (2012). Targeting oxidative stress component in the therapeutics of epilepsy. Curr. Top. Med. Chem..

[132] Fox C.K., Mackay M.T., Dowling M.M., Pergami P., Titomanlio L., Deveber G. (2017). Prolonged or recurrent acute seizures after pediatric arterial ischemic stroke are associated with increasing epilepsy risk. Dev. Med. Child Neurol..

[133] Choudhury G.R., Ding S. (2016). Reactive astrocytes and therapeutic potential in focal ischemic stroke. Neurobiol. Dis..

[134] Shlosberg D., Benifla M., Kaufer D., Friedman A. (2010). Blood-brain barrier breakdown as a therapeutic target in traumatic brain injury. Nat. Rev. Neurol..

[135] So E.L., Annegers J.F., Hauser W.A., O’Brien P.C., Whisnant J.P. (1996). Population-based study of seizure disorders after cerebral infarction. Neurology.

[136] Bryndziar T., Sedova P., Kramer N.M., Mandrekar J., Mikulik R., Brown R.J., Klaas J.P. (2016). Seizures Following Ischemic Stroke: Frequency of Occurrence and Impact on Outcome in a Long-Term Population-Based Study. J. Stroke Cerebrovasc. Dis..

[137] Bladin C.F., Alexandrov A.V., Bellavance A., Bornstein N., Chambers B., Cote R., Lebrun L., Pirisi A., Norris J.W. (2000). Seizures after stroke: A prospective multicenter study. Arch. Neurol..

[138] Waldbaum S., Patel M. (2010). Mitochondrial dysfunction and oxidative stress: a contributing link to acquired epilepsy?. J. Bioenerg. Biomembr..

[139] Iori V., Frigerio F., Vezzani A. (2016). Modulation of neuronal excitability by immune mediators in epilepsy. Curr. Opin. Pharmacol..

[140] Cavazos J.E., Cross D.J. (2006). The role of synaptic reorganization in mesial temporal lobe epilepsy. Epilepsy Behav..

[141] Risbud R.M., Porter B.E. (2013). Changes in microRNA expression in the whole hippocampus and hippocampal synaptoneurosome fraction following pilocarpine induced status epilepticus. PLoS ONE.

[142] Zhu X., Han X., Blendy J.A., Porter B.E. (2012). Decreased CREB levels suppress epilepsy. Neurobiol. Dis..

[143] Lin R.T., Cai R.R., Zhang P.F., Lin Y.X. (2016). Apoptosis and expression of Caspase 3 and Caspase 4 in neurocytes of refractory human temporal lobe epilepsy. Zhonghua Yi Xue Za Zhi.

